# Pulmonary Abnormalities in Animal Models Due to Niemann-Pick Type C1 (NPC1) or C2 (NPC2) Disease

**DOI:** 10.1371/journal.pone.0067084

**Published:** 2013-07-02

**Authors:** Blair R. Roszell, Jian-Qin Tao, Kevin J. Yu, Ling Gao, Shaohui Huang, Yue Ning, Sheldon I. Feinstein, Charles H. Vite, Sandra R. Bates

**Affiliations:** 1 Institute for Environmental Medicine, Perelman School of Medicine, University of Pennsylvania, Philadelphia, Pennsylvania, United States of America; 2 Department of Physiology, Perelman School of Medicine, University of Pennsylvania, Philadelphia, Pennsylvania, United States of America; 3 Department of Clinical Studies, School of Veterinary Medicine, University of Pennsylvania, Philadelphia, Pennsylvania, United States of America; International Centre for Genetic Engineering and Biotechnology, Italy

## Abstract

Niemann-Pick C (NPC) disease is due to loss of NPC1 or NPC2 protein function that is required for unesterified cholesterol transport from the endosomal/lysosomal compartment. Though lung involvement is a recognized characteristic of Niemann-Pick type C disease, the pathological features are not well understood. We investigated components of the surfactant system in both NPC1 mutant mice and felines and in NPC2 mutant mice near the end of their expected life span. Histological analysis of the NPC mutant mice demonstrated thickened septae and foamy macrophages/leukocytes. At the level of electron microscopy, NPC1-mutant type II cells had uncharacteristically larger lamellar bodies (LB, mean area 2-fold larger), while NPC2-mutant cells had predominantly smaller lamellar bodies (mean area 50% of normal) than wild type. Bronchoalveolar lavage from NPC1 and NPC2 mutant mice had an approx. 4-fold and 2.5-fold enrichment in phospholipid, respectively, and an approx. 9-fold and 35-fold enrichment in cholesterol, consistent with alveolar lipidosis. Phospholipid and cholesterol also were elevated in type II cell LBs and lung tissue while phospholipid degradation was reduced. Enrichment of surfactant protein-A in the lung and surfactant of the mutant mice was found. Immunocytochemical results showed that cholesterol accumulated in the LBs of the type II cells isolated from the affected mice. Alveolar macrophages from the NPC1 and NPC2 mutant mice were enlarged compared to those from wild type mice and were enriched in phospholipid and cholesterol. Pulmonary features of NPC1 mutant felines reflected the disease described in NPC1 mutant mice. Thus, with the exception of lamellar body size, the lung phenotype seen in the NPC1 and NPC2 mutant mice were similar. The lack of NPC1 and NPC2 proteins resulted in a disruption of the type II cell surfactant system contributing to pulmonary abnormalities.

## Introduction

Niemann-Pick disease type C (NPC) is an autosomal recessive lysosomal storage disorder marked by excess intralysosomal cholesterol, progressive neurodegeneration and hepatosplenomegaly that has no effective approved treatments [1]. Loss of NPC1 or NPC2 protein function results in Niemann-Pick type C1 or type C2 disease, respectively. The two types of NPC disorders are thought to be clinically and biochemically indistinguishable, with reports of pulmonary involvement in both with alveolar proteinosis and foamy macrophages, although NPC2 disease seems to have a more severe phenotype [2], [3], [4]. Whereas the pulmonary disease can be acute, the surfactant system in Niemann-Pick disease has not been fully described in detail. There has been a case report of a patient with a frameshift mutation in NPC2 and reduced NPC2 protein levels. The bronchoalveolar lavage fluid from this patient was analyzed and a marked accumulation of surfactant phospholipid and cholesterol was shown [2]. NPC2 hypomorph mice and patients with NPC2 disease develop alveolar periodic acid-Schiff-positive material, indicative of pulmonary alveolar proteinosis (PAP) [2]. PAP is a rare lung disorder characterized by abnormal accumulation of surfactant with little to no inflammation and fibrosis.

The most common mutations associated with NPC disease (95% of cases) are in the NPC1 gene with the affected patients demonstrating a broad clinical spectrum [5]. However, lung abnormalities are not well characterized. Animal models of NPC1 disease include mice and felines. The pathology of the lung in *Npc1^nih/nih^* mice demonstrated vacuoles in type I and endothelial cells and foamy alveolar macrophages [6]. Cholesterol and phospholipid accumulation in the lung organ of the *Npc1* mice has been documented but surfactant was not analyzed [6], [7], [8]. The feline model of NPC1 disease is a domestic short-haired cat with a spontaneous missense mutation (C955S) in NPC1. The feline NPC1 mutation is orthologous to the most common mutation in juvenile-onset patients [9]. The cats demonstrate progressive neurologic symptoms with dysmetria, ataxia and whole body tremor and the liver and spleens have significantly higher levels of unesterified cholesterol. Cultured feline fibroblasts demonstrate perinuclear accumulation of cholesterol utilizing the same assay employed in the identification of NPC disease in humans [10]. Pulmonary abnormalities have not been examined. The pathologic changes in the mouse and human lung with NPC disease implicate a dysfunction in type II cells, the primary surfactant secreting cell.

Pulmonary surfactant, produced by type II alveolar cells, performs the critical role of reducing surface tension in the alveoli. Cholesterol is the most abundant neutral lipid in surfactant, contributing 5–10% weight by volume, with the rest made up of phospholipids and protein [11]. Cholesterol influences the stability and surface viscosity of surfactant making it an important component in the determination of surfactant physical properties [12]. Elevated (>20%) cholesterol inhibits surfactant function, contributing to surfactant dysfunction in ARDS and acute lung injury [13], [14]. One model suggests that cholesterol accumulates at the surfactant-air interface, interfering with the formation of stacked bilayer patches, structures that contain surfactant lowering protein SP-C [15]. Turnover of surfactant phospholipid is well studied while little is known about the molecular mechanisms regulating surfactant cholesterol. Most lung cholesterol is derived from plasma lipoproteins and radioactive tracer studies demonstrated the delivery of labeled cholesterol from lipoproteins to lamellar bodies and, subsequently, to surfactant secreted into the alveolar space [16]. Type II pneumocytes synthesize and store surfactant in lamellar bodies (LBs), lysosome-related organelles, prior to secretion into the airspace. The Niemann-Pick C pathway has been shown to regulate the removal of low density lipoprotein (LDL)-derived cholesterol from the lumen of the late endosome/lysosome system to the cytosolic compartment [17], [18]. We hypothesized that this pathway may play a role in surfactant cholesterol transport since we showed that the Niemann-Pick C (NPC) proteins, NPC1 and NPC2 were present in lamellar bodies [19]. NPC1 is an endo-lysosomal transmembrane protein that contains a sterol-sensing domain [17]. NPC2 is a 132 amino acid soluble, mannose-6-phosphate targeted lysosomal protein that also is found in the alveolar space [18], [19]. A current model suggests that NPC2 binds the unesterified cholesterol released after the hydrolysis of LDL cholesteryl ester by lysosomal acid lipase. The NPC2 protein then docks to the second luminal domain of NPC1 and cholesterol is exchanged between NPC2 and the N-terminal domain of NPC1 [20], [21], [22]. NPC1 protein directs the egress of cholesterol from the lysosome through an unknown mechanism, possibly involving the oxysterol-binding protein-related protein 5 [23]. Inhibition of the NPC pathway in isolated type II cells with pharmacological inhibitor U18666A leads to accumulation of cholesterol in the lumen of lamellar bodies, suggesting that the NPC pathway plays a role in regulating lamellar body cholesterol [19].

Reports on the morphology of type II cells in lungs of patients or animal models of NPC deficiency describe the pneumocytes to be “unremarkable” [6]. However, due to the cholesterol accumulation in the lung tissue of NPC1-deficient mice [24] the marked pulmonary lipid accumulation in the NPC2 patient [2], and the presence of NPC1 and NPC2 in the lamellar bodies of type II cells [19], we hypothesized that mutations in either NPC1 or NPC2 would result in a dysfunction in pneumocytes and adversely alter the surfactant system. This study was undertaken to define the effects of disorders in cholesterol transport due to the absence of NPC proteins on type II cell homeostasis. We have employed detailed histological and biochemical analysis of alveolar type II cells, macrophages, lamellar bodies, and surfactant in NPC1 and NPC2 mutant mice and NPC1 mutant felines. The NPC1 and NPC2 pulmonary diseases were compared to determine whether any pathologic characteristics differed between them. As treatments for the central nervous system dysfunctions in NPC disease are making progress, a more complete understanding of the lung pathogenesis will aid in the assessment of new therapies specific for the pulmonary components of the disease.

## Materials and Methods

### Ethics Statement

The mice and felines were housed under the National Institutes of Health and USDA guidelines for the care and use of animals in research. The University of Pennsylvania Institutional Animal Care and Use Committee (IACUC) approved all experimental protocols.

### Animals

**Mice.** *Npc1^nih/nih^* and Npc2 hypomorph mice on a BALB/c background were obtained as a gift from Dr. Peter Lobel. Both types of mice are established models of NPC disease. The *Npc1* mouse defect arose due to a spontaneous mutation in mouse chromosome 18 [8]. 24 base pairs of unidentified sequence replaced 44 base pairs of the wild type sequence, resulting in a frame shift and premature truncation of the open reading frame [25]. The *Npc2* mutant mice were produced by Sleat, et al. and contain an aberrant recombination event such that the targeted allele has an additional repeat of intron 1 and replacement of amino acid cysteine 42 with a stop codon [26]. As a result, immuno-detection found little to no NPC2 expression in most tissues. The genotypes of the *Npc1* and *Npc2* mice were determined by PCR of genomic DNA from tail tips. The primer pairs have been previously described [26]. Mice were housed in a pathogen-free animal facility at the University of Pennsylvania in HEPA-filtered cages and were fed ad libitum. Both male and female mice were used in the study and were sacrificed between the ages of 9–13 weeks. Age-matched litter mates were used as wild type controls.

#### Feline

The felines were housed in the School of Veterinary Medicine of the University of Pennsylvania. NPC1 mutant diseased felines and their normal counterparts were produced from the same line. The NPC1 felines have a spontaneous missense mutation (C955S) in the NPC1 protein which results in lysosomal accumulation of unesterified cholesterol [9], [10]. At one day of age, peripheral blood leukocytes from blood drawn for the cats were tested by PCR analysis of genomic DNA to determine genotype [27]. Affected and control felines were used between 21 and 25 weeks of age.

### Type II Cell and Lamellar Body Isolation

The isolation of type II cells from mice lungs was performed as described previously [28]. Briefly, mice were anesthetized, the chest cavity exposed and the trachea was intubated with a mechanical ventilator to inflate the lungs. The heart was perfused in a saline solution to remove blood cells from the lung. Lungs were filled with saline, excised from the chest cavity, and washed three times with saline to remove macrophages and bronchoalveolar lavage (BAL). An elastase solution was instilled down the trachea, tissue was chopped in a McIlwain™ Tissue Chopper, enzyme was quenched in FBS, and cells were panned on a mouse IgG coated dish to remove macrophages. Later, non-adherent cells were moved to a separate dish and incubated overnight at 37°C. These cells were designated isolated type II cells.

Lamellar bodies were isolated as described previously [29]. Briefly, lungs of 2 to 6 mice or a piece of one lung of a cat were perfused with saline to remove blood cells, rinsed in 0.25 M sucrose, blotted on filter paper, homogenized, filtered through gauze, and pooled. The homogenate was separated on a sucrose gradient, with lamellar body bands appearing between 0.45 and 0.50 M sucrose. Lamellar body containing bands were removed with a glass pipette, the refractive index was measured with a refractometer and the molarity of the fraction was determined. The harvested fraction was diluted to 0.2 M sucrose in water, centrifuged at 20,000×g for 15 minutes. Pellets were stored at −80°C until use.

### Western Blotting

Lung tissue from 9–14 week old mice was collected, homogenized, and separated using denaturing sodium dodecyl sulfate-polyacrylamide gel electrophoresis as previously described (8). Primary antibody against rabbit anti-NPC1 (diluted 1∶200 Novus Biologicals, Littleton, CO), NPC2 (diluted 1∶750, Sigma, St. Louis, MO, Cat# HPA000835), secondary antibody goat anti-rabbit IgG horseradish peroxidase-conjugated (diluted 1∶3,000, Upstate Millipore, Billerica, MA), and ECL Plus Western Blotting Detection Reagent (GE Healthcare, Amersham, Piscataway Township, NJ) were used.

### Histology & Alveolar Septal Thickness

Lungs from wild type, NPC1 and NPC2 mutant mice were excised from 3 animals for each experimental group. From each mouse, the left lobe, upper right lobe, and lower right lobe were fixed overnight in a mixture of formaldehyde and glutaraldehyde, and embedded in paraffin. Tissue was cut into 5 µm sections and stained with hematoxylin and eosin. Random fields of distal airway were collected at 60× magnification. Alveolar septal thickness was determined using an overlaid test grid. The alveolar septal thickness was measured as the length of a grid line that extended from one alveolus to an adjacent alveolus. Regions containing large airways or capillaries were not included in this measurement. >200 probe “hits” of the alveolar septal thickness were gathered from more than 5 sections per mouse and 15 fields for each experimental condition.

### Electron Microscopy

Lungs from mice were perfused through the pulmonary artery with 0.1 M sodium cacodylate buffer to remove blood cells. Lungs were excised and fixed by perfusion with 5% glutaraldehyde through the pulmonary artery and trachea. The trachea was removed, and whole lungs were fixed for at least 4 hours. A portion of the lung from a cat were perfused with cacodylate buffer through the bronchi and fixed by immersion in 5% glutaraldehyde**.** Lungs were cut into 1 mm^3^ blocks, treated for 2 hours with 2% osmium, dehydrated, embedded in EPON, and polymerized for 48 hours at 60°C. Blocks were sliced into ultrathin (80 nm) sections in a Leica Ultracut UCT ultramicrotome. Samples were stained with 2% uranyl acetate and 0.5% lead citrate. All samples were imaged on a TEM (100-CX, Jeol).

### Measuring Cholesterol and Phospholipid

To measure cholesterol, samples were prepared following instructions in the Amplex Red Cholesterol Assay Kit (Molecular Probes, Eugene, OR, Cat #A-12216). Protein for each corresponding sample was determined using the Lowry assay and data are presented as µg lipid/µg protein [30]. Phospholipid concentration was determined by phosphorus assay [31]. Briefly, the lipid fraction was separated from the water soluble fraction using the Bligh-Dyer method and dried completely in a glass tube [32]. Dried samples were resuspended in 70% perchloric acid (HClO_4_), heated for 20 minutes, and then cooled. Distilled water and ammonium molybdate were added to the sample and vortexed before the addition of reagent A (amino naphtha sulfonic acid, sodium sulfite, Na meta-Bisulfite). Samples were boiled for 10 minutes, cooled and colorimetric reading was done at 830 nm.

### Isolated Lung Perfusion

Uptake and degradation of liposomes labeled with [choline-methyl-^3^H] dipalmitoyl phosphatidylcholine ([^3^H]-DPPC) by the isolated, perfused lung was performed as described previously [33]. Briefly, liposomes of DPPC, egg phosphatidylcholine (PC), egg phosphatidylglycerol, and cholesterol (molar ratio of 10∶5:2∶3) with trace amounts of [^3^H]-DPPC were prepared by freezing and thawing followed by extrusion under pressure through a 100-µm pore-size filter. The liposomes (10 nmol of DPPC in 20 µl of saline) were instilled into the lungs of anesthetized mice; the lungs were removed and placed in a perfusion apparatus under ventilation. After 5 min (time required for lung isolation, baseline) or 2 hours, the lungs were lavaged and the lung tissue homogenized. The homogenate was extracted [32] and the aqueous and lipid fractions isolated. Phospholipids in the organic phase were fractionated by thin layer chromatography on silica gel plates with chloroform-methanol-ammonia-water (65∶35:2.5∶2.5, vol/vol) as the solvent system and bands of interest scraped and counted. Disaturated phosphatidylcholine (DSPC) was separated from total PC on a neutral alumina column after osmication of lipids [34]. Unsaturated PC, the product of lysoPC reacylation, was calculated as total dpm in PC minus dpm in disaturated PC (DSPC). Total degradation of internalized DPPC was calculated from the sum of dpm in lysoPC, aqueous, and unsaturated PC after subtraction of the baseline data (5 min).

### Immunocytochemistry

Type II cells were fixed in 4% paraformaldehyde for 20 minutes. Cells were washed, fixative was quenched in 0.3% glycine, and cells were permeabilized in 0.2% triton for 30 minutes at room temperature. Cells were blocked in 10% BSA with 2% normal goat serum for 1 hour. Primary antibodies were incubated on cells overnight in dilute blocking buffer. The next day, cells were washed and incubated in secondary antibody for 1 hour. Cells were then washed and imaged.

### Statistics

All data are expressed as mean ± SEM. Statistical comparisons were performed using SigmaStat (Systat Software, Inc.). Data were analyzed using a Student’s *t*-test or ANOVA. Results were considered statistically significance at *P*≤0.05.

## Results

**Figure 1:**
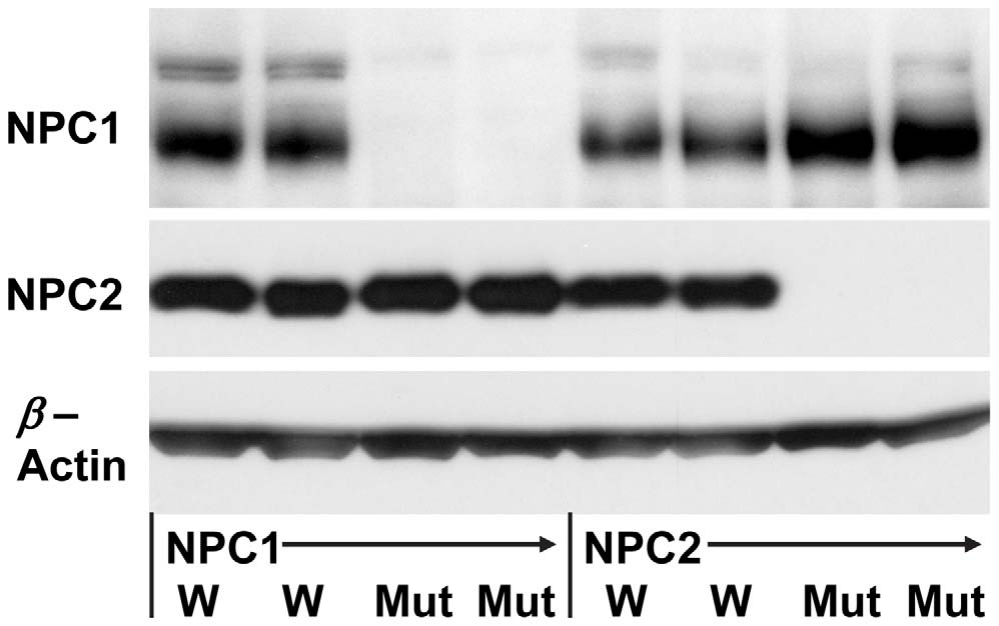
NPC proteins in mouse lungs. Western blot of wild type (W) littermates, NPC1 (Mut) or NPC2 (Mut) mutant mouse lungs using anti-NPC1 or -NPC2 antibody. β-actin used as a loading control. 30 µg protein/lane.

NPC1 and NPC2 mutant mice have average life spans of 72 days [35] and 100 days [26], respectively. We examined the lung tissue of NPC1 and NPC2 mutant mice by Western blot in homozygous mutant animals near the end of their lifespan (approximately 63–70 days and 84–89 days, respectively). As shown in Fig. 1, there was no NPC1 or NPC2 protein expression detected in the lung tissue of the respective NPC1 and NPC2 mutant mice kept on a BALB/c background.

### Lung Morphology

**Figure 2:**
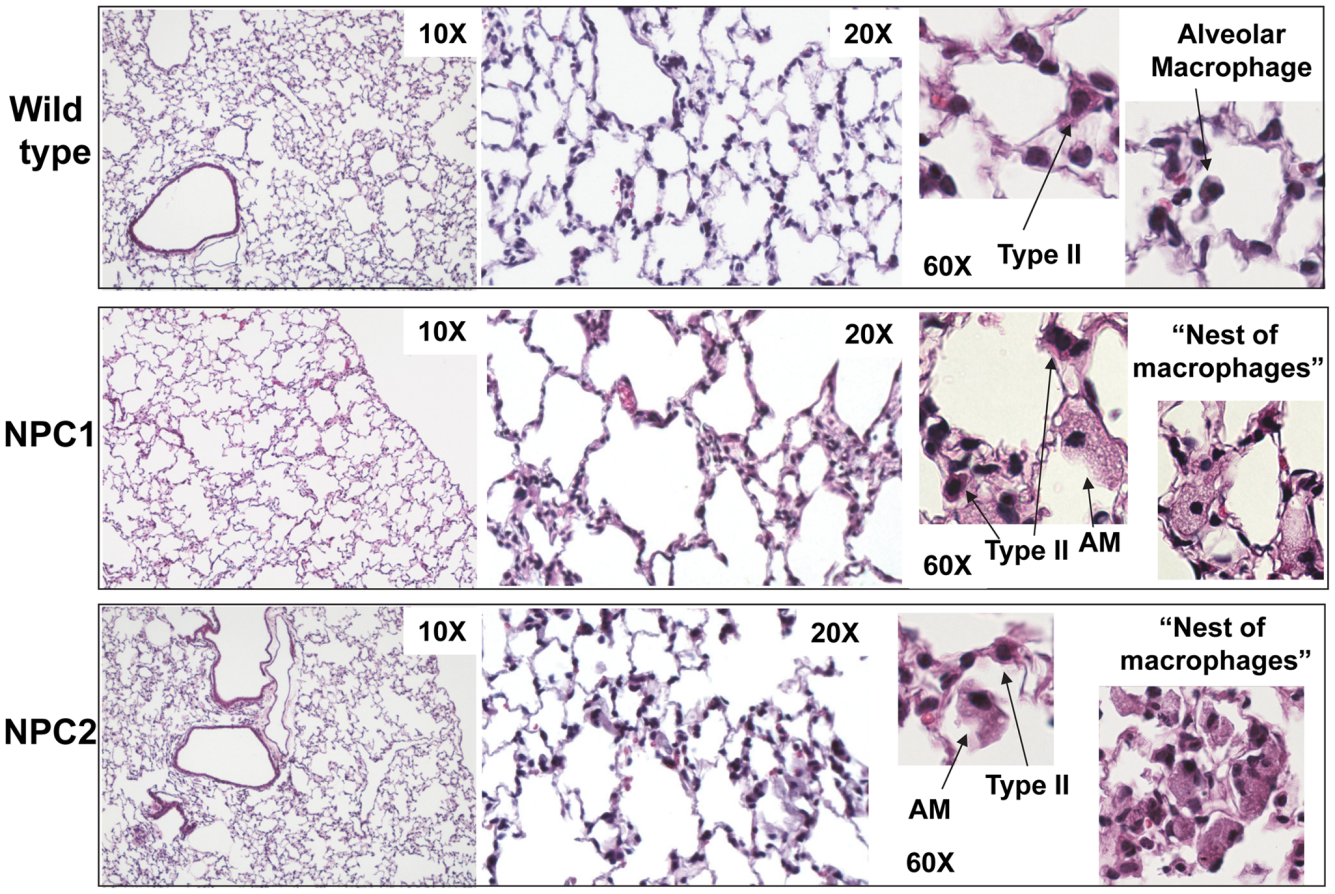
Light micrograph of mutant mice lungs stained with hematoxylin and eosin. Lungs from BALB/c wild type (95 days), NPC1 mutant (70 days) and NPC2 mutant mice (88 days). NPC1 and NPC2 mutant mice show “nests” of macrophages and alveolar macrophages with large inclusions.

Histological evaluation of the lungs of NPC1 and NPC2 mutant mice using hematoxylin/eosin stained sections was performed to determine pulmonary effects due to loss of cholesterol transport. The micrographs showed a thickening of the intra-alveolar septae with a slight enlargement of the airways in the NPC1 mice (Fig. 2). Quantitation of alveolar septum thickness revealed a significant (P<0.05) increase in average thickness in NPC1 (9.1±0.3 µm, n = 298) and NPC2 (10.8±0.5 µm, n = 401) mutants compared to wild type mice (5.8±0.2 µm, n = 421). Both types of NPC mutant mice lung contained “nests” of vacuolar filled macrophages and enlarged foamy alveolar macrophages as previously reported for NPC1 mice [6]. At this level, type II cells appeared unremarkable.

**Figure 3:**
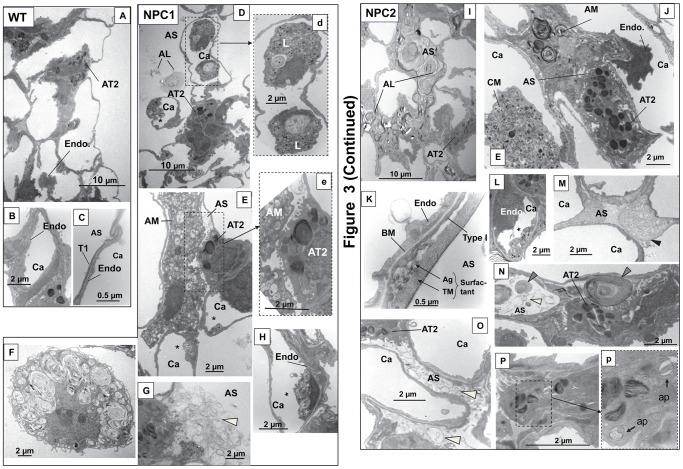
Morphology of wild type and mutant mice lungs by electron microscopy. Wild type lung (A–C). A. Overview of section of lung with alveolar type II cell (AT2) and endothelial cell (Endo). B. Capillary (Ca). C. Respiratory membrane with type I cell (T1) and endothelial cell (Endo). AS, alveolar space. NPC1 mutant lung (D–H). D. Overview of lung with leukocytes in the capillary and excess surfactant in alveolar space. AL, alveolar lipidosis. d. Enlargement of area in D showing vacuolar leukocyte (L). Similar leukocytes were seen in NPC2 mutant lung. E. Alveolar type II cell (AT2) with a foamy alveolar macrophage (AM) in close proximity in the alveolar space. e. Enlargement of area in E showing type II cell-macrophage contact. *Indicates vacuolar inclusions in endothelial cell. F. Alveolar macrophage with lipid-like material and vacuolar inclusions. G. Type II cells with excess surfactant (white arrowhead). H. Endothelial cell with vesicular inclusions (*). NPC2 mutant lung (I–P). I. Overview of lung with surfactant completely filling the alveolar space characteristic of alveolar lipidosis. J. AT2 with an alveolar macrophage containing multivesicular whirls and a foamy circulating macrophage (CM). K. Respiratory membrane of endothelial cell, basement membrane (BM) and type I cell and demonstrating large amounts of surfactant as tubular myelin (TM) and aggregate (Ag) structures. L. Endothelial cell with vesicular structures. M. Alveolar space with black arrowhead indicating proteinaceous material. Similar material was seen in NPC1 mutant lung. N. Large aggregate structure with tightly packed phospholipid-type whirls (gray arrowheads) or string-like structures (white arrowhead) in alveolar space. O. Surfactant vesicles (white arrowheads) filling the alveolar space. P. Type II cell with inset (p) showing autophagosome-like structures (ap) in enlargement.

Upon closer inspection of the alveolar architecture at the electron microscopic level, more pronounced pulmonary abnormalities were apparent. In Fig. 3, the wild type (WT) lung demonstrated a normal appearing type II cell (A) and capillary (B) and no macrophages (A) or visible surfactant (C). In contrast, within the NPC1 and NPC2 mouse lungs (Fig. 3, NPC1, D and NPC2, I) there was surfactant accumulation, a characteristic of alveolar lipidosis (AL). The severe, more pronounced lipid accumulation seen in NPC2 mice (Fig. 3, NPC2, I), although present in both types, was not as typical as the examples shown in Fig. 3, NPC1, D or NPC2, O. A detailed assessment of the surfactant accumulation in these mouse models revealed several physical forms which ranged from the striking accumulation of surfactant as tight or loosely packed whirls of phospholipid-like surfactant material in focal areas of the alveolar spaces as seen in the NPC2 mutant mice (Fig. 3, NPC2, N) to the most common form, string-like surfactant (Fig. 3, NPC1, D and G and NPC2, N and O). In addition there was an accumulation of tubular myelin and surfactant aggregates in large airways (Fig. 3, NPC2, K) and large surfactant aggregates next to type II cells or in the alveolar space (Fig. 3, NPC2, N). Proteinaceous-like material in the alveolar space also was found in the lungs of both mice (example in Fig. 3, NPC2, M).

Within mouse lung capillaries were enlarged polymorphonuclear leukocytes or circulating macrophages filled with vacuolar inclusions were found in both mutant mice, apparent in NPC1 mice Fig. 3D, as reported previously [6], and NPC2 mice (Fig. 3, NPC2, J). Capillary endothelial cells containing enlarged vacuoles/multivesicular bodies (designated by asterisks) were found in both NPC1 (Fig. 3, NPC1, E,H) and NPC2 (Fig. 3, NPC2, L) lungs, as previously described [6] in NPC1 mice. In both NPC mutant mice, alveolar macrophages appeared greatly enlarged and vacuole-filled, some with concentric multilamellar electron dense surfactant-like materials (Fig. 3, NPC1, F, for example). Alveolar macrophages were often found in close contact with type II cells (Fig. 3, NPC1, E and NPC2, J). The type II cells were marked by the presence of many autophagosomes found in both types of NPC mutant mice (Example from an NPC2 mutant mouse in Fig. 3, NPC2, P).

### Size of Type II Cell Lamellar Bodies

**Figure 4:**
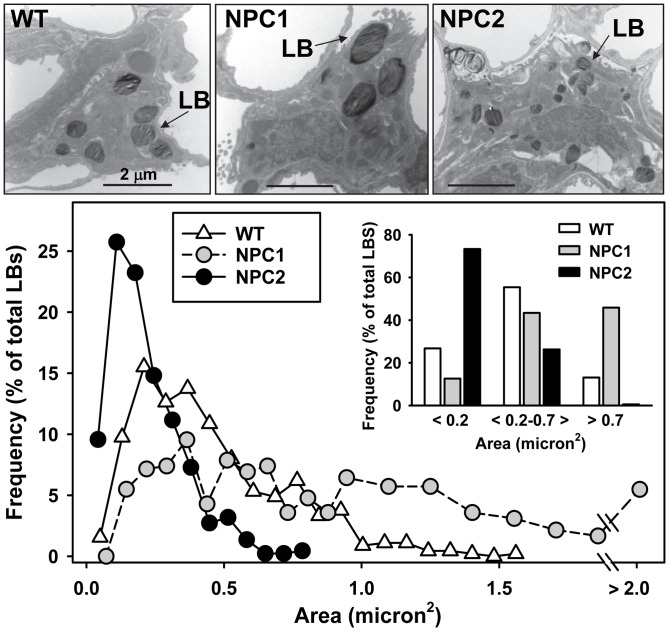
Size of mouse lamellar bodies. Using electron microscopic photographs of type II cells from wild type, NPC1 and NPC2 mutant mice, the size of the lamellar bodies was analyzed using ImageJ. Top. Electron micrographs of typical type II cells from wild type (WT, left), NPC1 (middle) and NPC2 (right) mutant mice. LB, lamellar body. Bottom. Histogram of the lamellar bodies from wild type (white triangles), NPC1 (gray circles) and NPC2 (black circles) type II cells. Frequency of each lamellar body size in micron^2^ is expressed as a % of the total numbers of lamellar bodies. Inset: size of lamellar bodies in grouped bins. Wild type, 60 type II cells, 451 LBs, 3 mice; NPC1, 53 type II cells, 435 LBs, 3 mice; NPC2, 37 type II cells, 459 LBs, 3 mice. *Statistically significant difference, P<0.05. E. Lamellar body sizes in grouped bins of ranges of areas.

It appeared that the lamellar bodies of type II cells from the affected mice differed in size. Thus, a morphometric analysis of lamellar body size of type II cells was performed for the NPC1 and NPC2 mutant lungs and their matching litter mates using electron microscopic images of the type II cells and ImageJ analysis. The size of the LBs of wild type mice were primarily distributed in the range from 0.2 µm^2^ to 0.7 µm^2^. While some LBs in NPC1 mutant mice showed a distribution similar to wild type, a significant proportion of NPC1 type II cell LBs were enlarged (Fig. 4). 42% of NPC1 mutant LBs had an area of 0.7 µm^2^ or larger, compared to only 15% of wild type LBs. Surprisingly, most of the LBs of the NPC2 mutant mice (70%) were similar in size as those of the smaller sized wild type LBs (<0.2 µm^2^) and were fairly uniform with none larger than 0.7 µm^2^. The mean average size of the LBs of the mice reflected these differences with NPC1 (0.81±0.02 µm^2^)>WT (0.43±0.02 µm^2^)>NPC2 (0.22±0.01 µm^2^), all statistically significantly different (P<0.001, n = 435–451 LBs) from each other. Interestingly, compared to the number of LBs per cell in wild type alveolar type II cells (WT: 7.5±0.7 LBs/cell, 60 cells), NPC2 alveolar type II cells exhibited 65% more LBs (NPC2∶12.4±1.1 LBs/cell, 37 cells, p<0.001) while the LB numbers/cell between WT and NPC1 were approximately the same (NPC1∶8.2±0.6 LBs/cell, 53 cells).

### Abnormal Lipid and Surfactant Protein-A (SP-A) Content of NPC Mutant Mouse Lung

**Figure 5:**
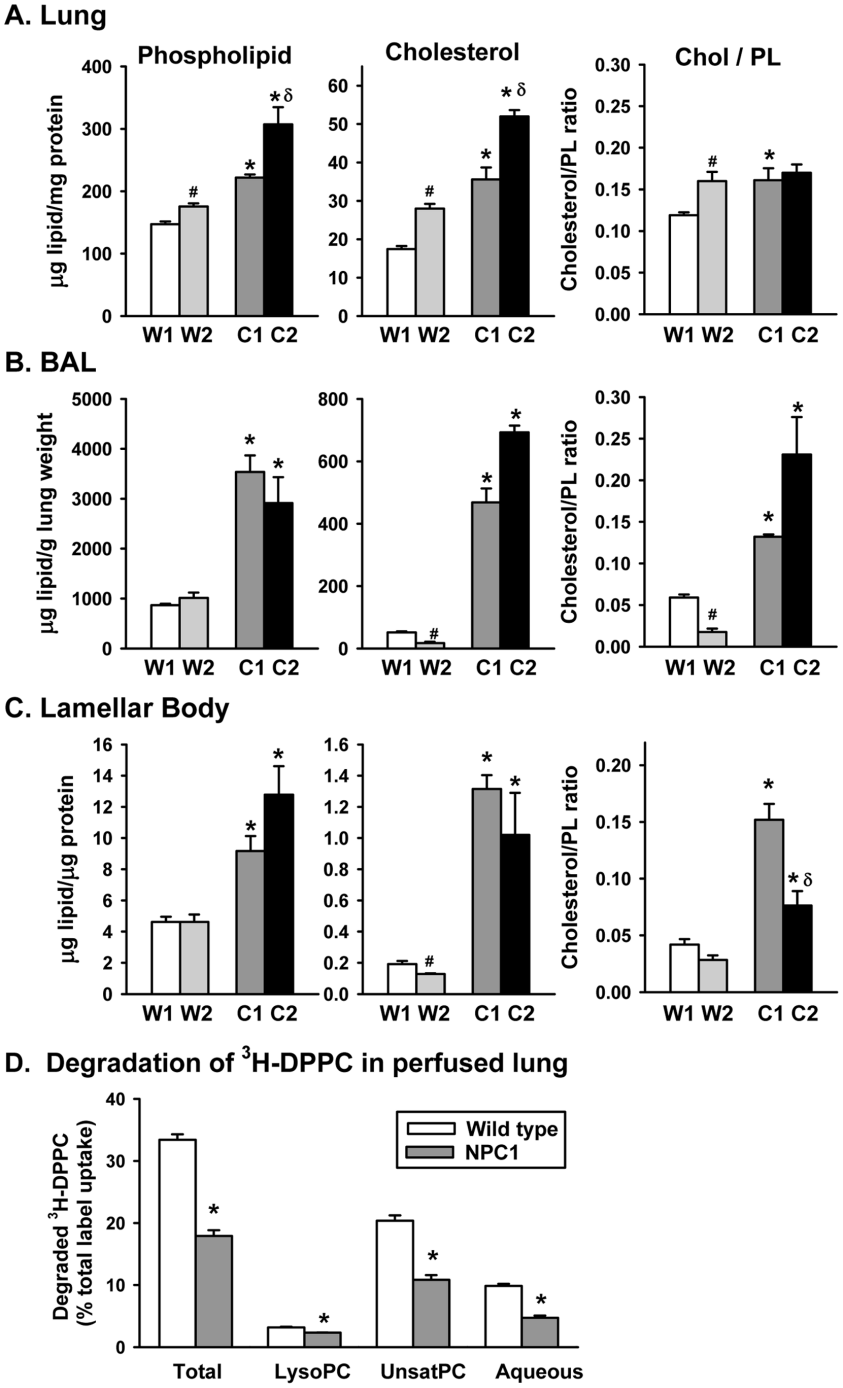
Lipid content and degradation of DPPC in mice lungs. A–C Cholesterol and phospholipid content of mice lungs. A. Lipid content of the lavaged lung as µg lipid/mg lung protein. Data are mean±SE, n = 4 separate mice. B. Broncho-alveolar lavage (BAL) of mice lungs. Data as µg lipid/gm weight of lung and are mean±SE, n = 4 separate mice. C. Lipid content of lamellar bodies isolated from the lungs of the wild type and mutant mice. Four (NPC1 or wild type littermates) or three (NPC2 or wild type littermates) lamellar body preparation isolated from the lungs of 2–6 mice. *Statistically significant difference versus wild type littermates, ^#^statistically significant difference between wild types. δ Statistically significant difference between mutants, *P*<0.05. D. Degradation of ^3^H-labeled DPPC as percentage of total label uptake. ^3^H-DPPC liposome degradation by the isolated, perfused lungs of three wild type or NPC1 mutant mice after intratracheal instillation. Values are means±SE. *Statistically significant difference from wild type. P<0.001, n = 3. Total degradation is the sum of lysophosphatidyl choline (lysoPC), unsaturated PC (unsatPC), and the aqueous fractions.

An enrichment in the cholesterol content of NPC1 mouse lungs has been documented previously [24]. The surfactant system of both the NPC1 and NPC2 mice was examined specifically by measuring changes in the phospholipid and cholesterol levels of the lung tissue, surfactant and lamellar bodies. Measurements of the physical parameters of the mice used for the analysis presented in Fig. 5 are shown in Table 1 and were typical for all the mice in the study. The mutant mice had lower body weights than their age-matched littermates. Since the lung weights were similar between groups, the lung weight as a % of total body weight was higher in the NPC mice (Table 1), as was shown previously for NPC1 mice [8]. The lungs of the mice were lavaged and the surfactant and lavaged lung tissues were processed for lipid and protein content. Lipid analysis of the lungs of the wild type mice demonstrated a minor enrichment of phospholipid and cholesterol in the wild type littermates of the NPC2 mutant mice compared to the littermates of the NPC1 mice, possibly due to the age differences (89 vs. 63 days, respectively) (Fig. 5); however, the lungs from both mutant mice showed a significantly elevated lipid content over littermate controls. Both the phospholipid and cholesterol content of the lavaged lung tissue from the NPC1 and NPC2 mutant mice were enriched over their respective controls, as has been shown previously for the cholesterol [8], [24] and phospholipid [8] content of NPC1 mutant mice where the entire lung as an intact organ was examined.

**Table 1:** Physical parameters of NPC mice and wild type littermates.

Genotype	Body weight (gm)	Lung weight (mg)	Lung wt. as % of body wt.	Age (days)	BAL protein (µg/mg lung wt.)	n
Wild type, 1	23.7±0.7	163.2±1.4	0.69±0.03	63±1	1.60±0.12	4
NPC1	14.2±0.5*	145.2±2.6*	1.03±0.04*	63±1	2.99±0.23*	4
Wild type, 2	21.4±2.1	160.9±7.8	0.76±0.04	89±4#	1.38±0.15	4
NPC2	13.2±1.2*	148.8±4.2	1.14±0.07*	89±3#	2.00±0.37	4

Data are mean±SEM. Wild type, 1, indicates wild type corresponding to NPC1 mice; Wild type, 2, indicates wild type corresponding to NPC2 mice; wt, weight; n, number of mice.

* Statistically significantly different from wild type.

# Statistically significantly different from NPC1 affected or wild type, 1 mice.

As would be predicted from the lipidosis in the alveolar space observed in the electron microscopic data, there was a marked increase in surfactant phospholipid in both mutant mice. The phospholipid levels of the bronchoalveolar lavage (BAL) were elevated over control mice in the NPC1 mutant (4-fold) and in NPC2 mutant mice (3-fold) (Fig. 5B), mirroring the abnormal accumulation of surfactant within the alveoli at the EM level (Fig. 3, NPC1, G, and NPC2, I). Even more striking was the elevation in cholesterol levels, 12-fold in NPC1 (12-fold) and 35-fold in NPC2 mutant BAL (Fig. 5B). When compared as a ratio, the cholesterol to phospholipid ratio was increased in both NPC1 and NPC2 mutant BAL compared to wild type (Fig. 5B).

**Figure 6:**
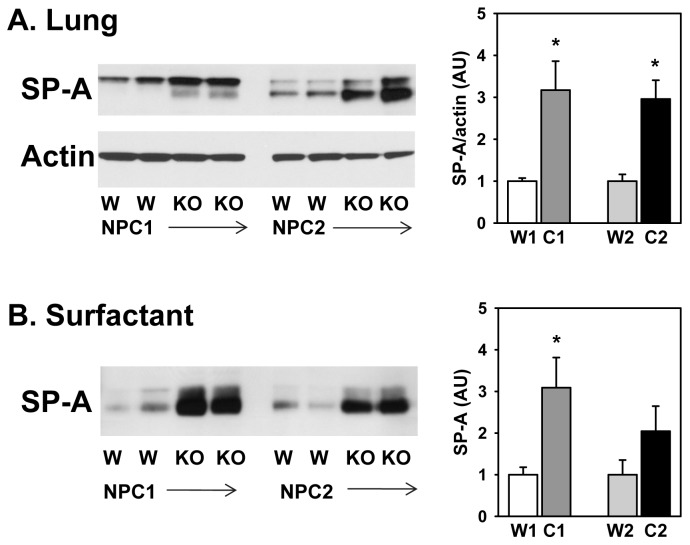
Surfactant protein-A (SP-A) content of (A) lungs and (B) surfactant isolated from wild type (W) or NPC mutant (NPC1, NPC2) mice. Left, Western blots of SP-A or actin. Right, quantitation of Western blots. A. Arbitrary units (AU) of SP-A relative to actin from lungs of NPC1 or NPC2 mutant mice (C1 or C2) or age-matched wild type controls (W1 or W2) (n = 6–8) or B. Arbitrary units of SP-A in surfactant (n = 4–9). All samples were loaded at equal protein values. *Significant difference from wild type, (*P*<0.05). The two SP-A bands are due to differences in glycosylation.

Analysis of the bronchoalveolar lavage showed that the NPC1 mutant mouse lung had a mild protein accumulation which was not the case for the NPC2 mutant mice (Table 1). We further characterized the lungs of the mutant mice, by analyzing the levels of SP-A, the most abundant surfactant protein. Shown in Fig. 6 is the quantitation (in arbitrary densitometry units) of the amount of SP-A in the lung (normalized to actin) or in the surfactant (with equal protein loading) and expressed relative to wild type samples run on the same gel. SP-A levels were elevated three-fold in the lungs and surfactant of the NPC1 mutant mice compared to age-matched wild type. A similar enrichment in SP-A was seen in the lungs of the NPC2 mutant mice. In the NPC2 surfactant, SP-A levels were enhanced but the change did not reach statistical significance.

Lipid analysis was performed on lamellar bodies isolated from pooled samples of lung homogenates, either from NPC1 mutants, NPC2 mutants or the wild type counterparts. The data revealed an increase in phospholipid (2 to2.8-fold increase in both animals) and cholesterol (6.8-fold increase and 7.9-fold increase, respectively) content compared to wild type animals (Fig. 5C), with a greater increase in cholesterol as shown by the cholesterol to phospholipid ratio. Overall, changes in lung lipid content were comparable between the NPC1 and NPC2 mutant mice, with relatively minor differences.

To determine the effect of lipid enrichment of the lung and lamellar bodies on surfactant uptake and degradation, we utilized the isolated, perfused lung system. ^3^H-choline-labelled DPPC liposomes were instilled into the lungs of wild type or NPC1 mutant mice. After a two hour perfusion time, there was no significant difference in the endocytosis of the DPPC by the lungs (5.5±0.4% for wild type vs. 5.3±0.3% for NPC1, n = 3). However, total degradation of ^3^H-DPPC was reduced by 46% in the NPC1 mice lungs as compared to wild type due to significant decreases in all lipid and aqueous fractions (Fig. 5D). Thus, the lipid accumulation and the absence of NPC1 protein resulted in a decrease in DPPC degradation.

### Cholesterol Accumulation in Lamellar Bodies of Type II Cells of NPC1 Mutant Mice

**Figure 7:**
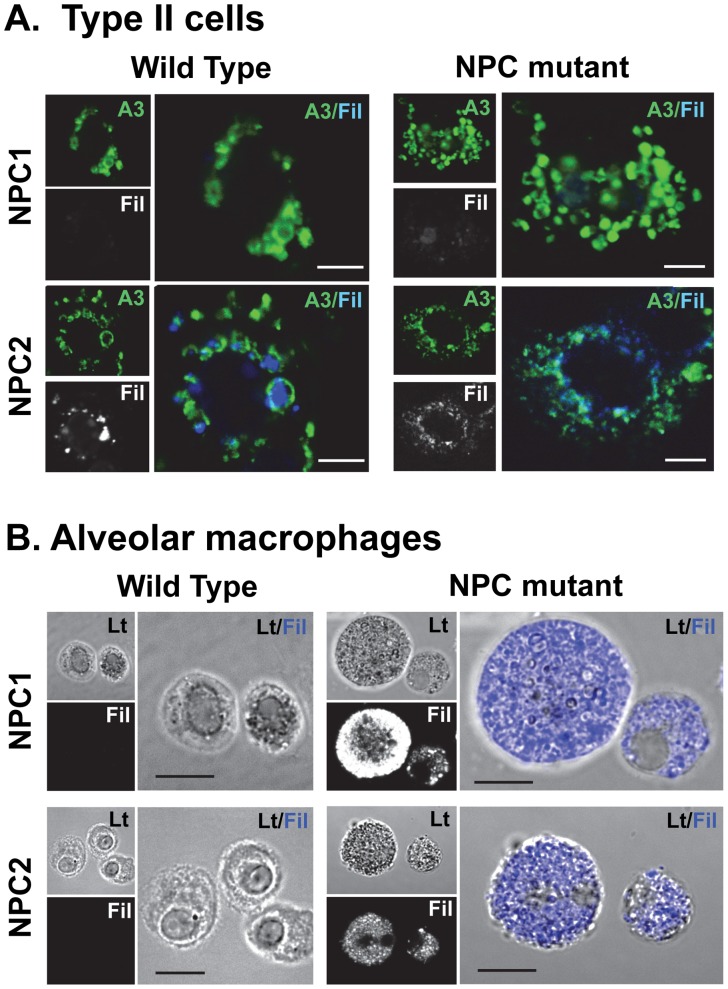
Immunocytochemistry of (A.) type II cells or (B.) alveolar macrophages isolated from wild type and NPC mutant mice. A. Type II cells were isolated from NPC1 (Top) or NPC2 (Bottom) mutant mice or their corresponding wild type littermates and placed in culture for 24 hrs. The cells were fixed and stained with anti-ABCA3 antibody (green) to mark the lamellar body limiting membrane or filipin (Fil, gray or blue) to mark cholesterol. The merged pictures with anti-ABCA3 in green and filipin in blue are enlarged. Scale bar = 5 µm. **B**. Alveolar macrophages from NPC mutant mice contain cholesterol. Alveolar macrophages were isolated from the lung lavage from NPC1 (Top) or NPC2 (Bottom) mutant mice or their corresponding wild type littermates and placed in culture for 2 hrs. The cells were fixed and stained with filipin which labels free unesterified cholesterol. Lt, Phase micrograph; Fil, Filipin stain in gray. Merge of phase and filipin (blue) are enlarged. Scale bar = 10 µm.

To validate the biochemical finding of excess cholesterol in lamellar bodies, we examined the cellular distribution of cholesterol in isolated alveolar type II cells using filipin, a fluorescent, free cholesterol-binding probe (Fig. 7A). In NPC1 mutant type II cells, free cholesterol was apparent within the lumen of lamellar body structures, marked by ATP-binding cassette sub-family A member 3 (ABCA3). NPC2 mutant type II cells had evidence of filipin staining of ABCA3 positive vesicles but the smaller size of the lamellar bodies made intravesicular localization difficult (Fig. 7A).

### Alveolar Macrophages

**Figure 8:**
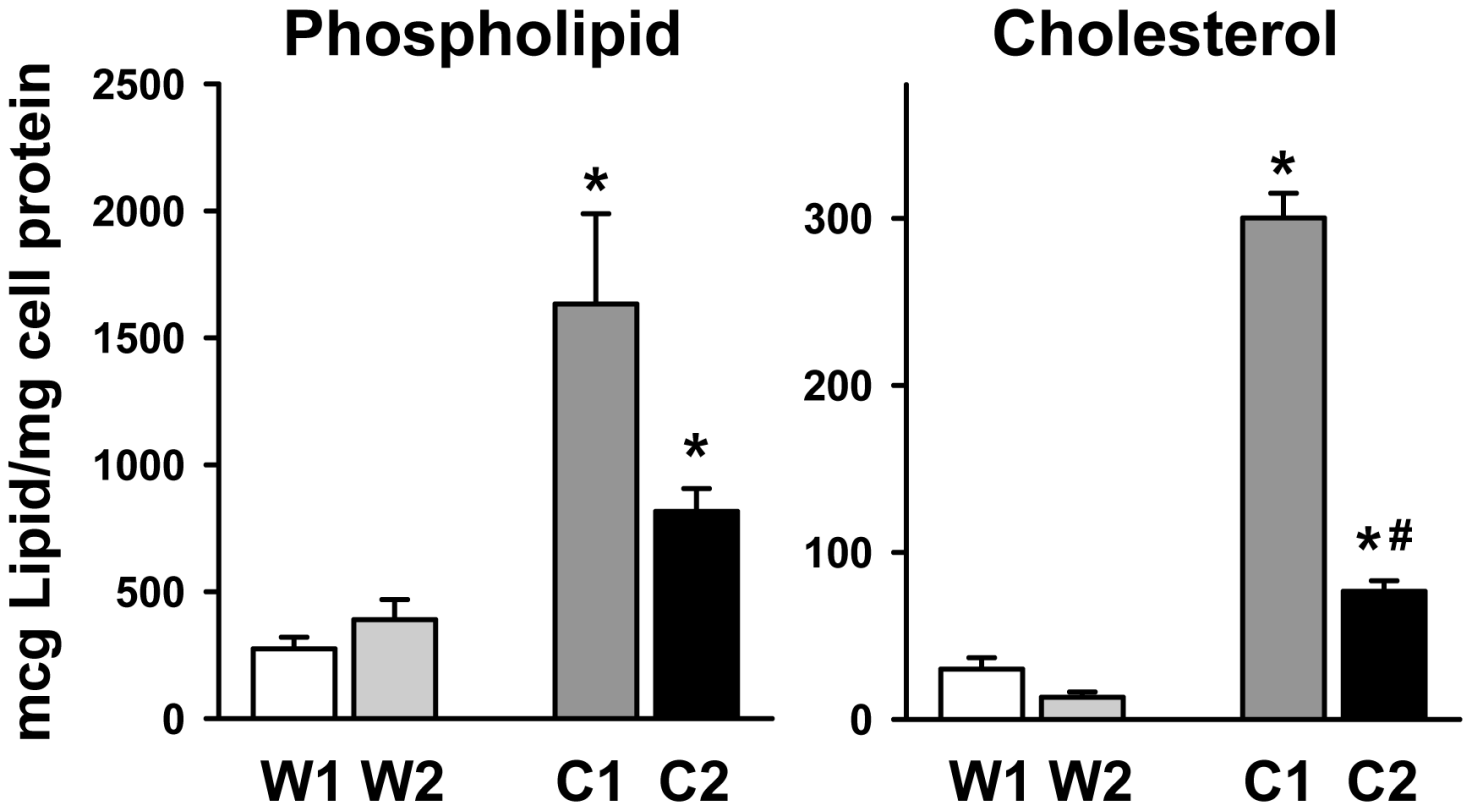
Lipid content of NPC mutant macrophages is elevated. Alveolar macrophages were isolated and cultured as in Fig. 7. The cells were harvested and the phospholipid and cholesterol content analyzed. W1,W2. Wild type littermates from NPC1 or NPC2 mutant mice, respectively. C1, NPC1; C2, NPC2. The data are mean±SE, n = 4 separate mice. *Statistically significant difference from corresponding wild type littermates. ^#^Significant difference between NPC1 and NPC2 mutant macrophages, P<0.05.

Alveolar macrophages and type II cells together are responsible for the majority of surfactant turnover, therefore we examined alveolar macrophages from NPC1 and NPC2 mutant mice [36]. Alveolar macrophages isolated from BAL of NPC1 and NPC2 mutant mice were laden with free cholesterol with many large, foamy cells (Fig. 7B) compared to wild type macrophages. We measured lipid levels in isolated alveolar macrophages and found that phospholipid levels were elevated 6-fold in NPC1 mutant mice, and 2-fold in NPC2 mutant mice. Cholesterol was also elevated 15-fold in NPC1 mutant macrophages and 8-fold in NPC2 mutant macrophages (Fig. 8).

### Feline NPC1 Disease

**Figure 9:**
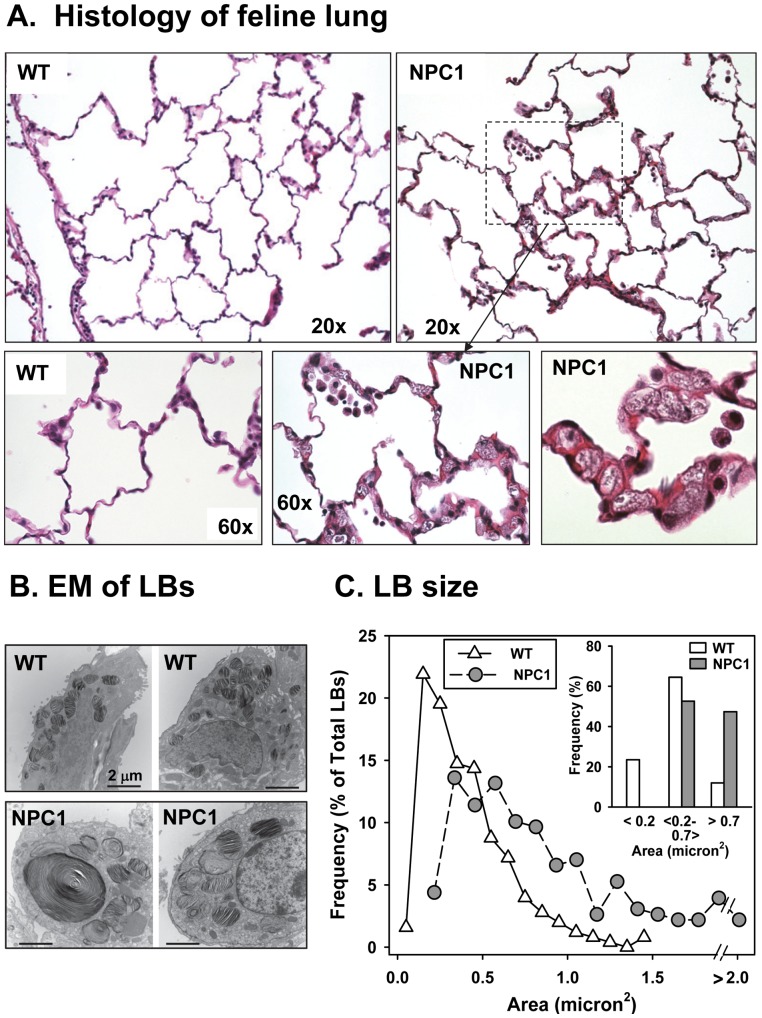
Micrograph of feline lung. A. Histology of feline lung. Wild type (WT) and NPC1 mutant (NPC1) feline lungs (25 weeks old) stained with hematoxylin and eosin. NPC1 mutant feline show thickened septae, enlarged “foamy” macrophages in capillaries and alveolar macrophages in the alveolar space. **B.** Electron micrographs (EM) of typical type II cells from wild type (WT) and NPC1 mutant (NPC1) feline indicating that the size of NPC1 mutant feline lamellar bodies is enlarged. **C**. Histogram of the lamellar bodies from wild type (white triangles) and NPC1 (gray circles) type II cells. Using electron microscopic photographs of type II cells from wild type and NPC1 mutant felines, the size of the lamellar bodies was analyzed using ImageJ. **C**, Inset: size of lamellar bodies in grouped bins in ranges of areas. Frequency of each lamellar body size in micron^2^ is expressed as a % of the total numbers of lamellar bodies. Wild type (WT): 28 type II cells, 250 LBs, 3 feline; NPC1 mutant: 35 type II cells, 323 LBs, 3 feline.

**Figure 10:**
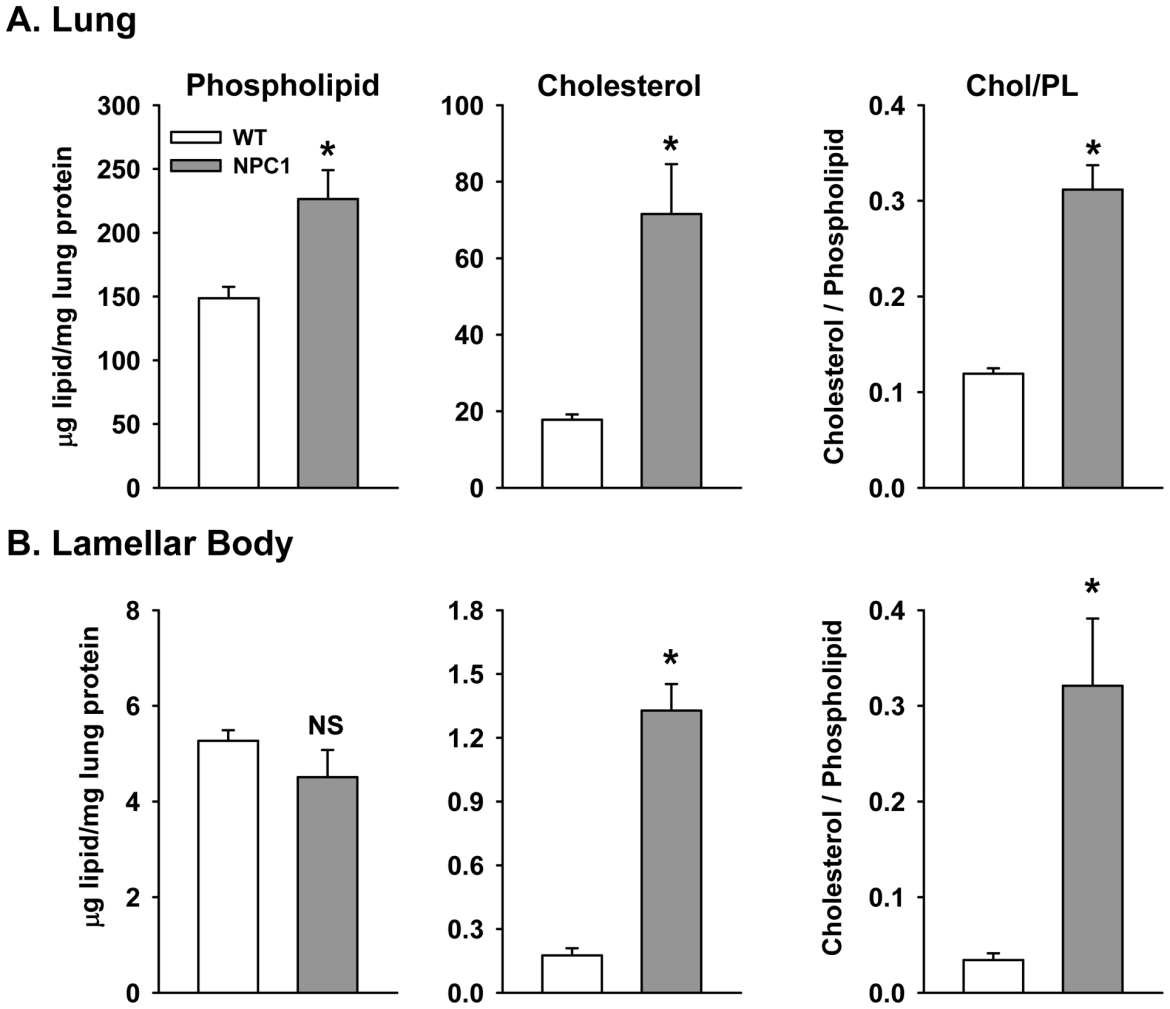
Phospholipid and cholesterol content of feline lungs. A. Lipid content of the lung. Data are mean±SE, n = 3 feline. B. Lipid content of lamellar bodies isolated from the lungs of the wild type and NPC1 mutant feline. Data are the mean±SE and range of 5 (WT) or 4 (NPC1 mut) lamellar body preparations isolated from feline lungs analyzed in triplicate. *Significant different from WT.

The disease in affected NPC1 mutant cats is phenotypically, morphologically, and biochemically similar to patients with a mutation in the NPC1 gene and less acute onset [10]. Lung histology revealed thickened septae, foamy leukocytes in the capillaries and evidence of white blood cell infiltrate (Fig. 9) in the affected feline. Analysis of the size of the feline lamellar bodies using ImageJ quantitation of electron micrographs demonstrated a range of sizes surprisingly similar to that seen with mice (Fig. 10). The area of most of the wild type lamellar bodies ranged between 0.2 and 0.7 micron^2^, while most of the NPC1 mutant feline lamellar bodies were larger than 0.5 micron^2^. Similar to the mice lamellar bodies, the mean size of the NPC1 mutant mice lamellar bodies were 0.90±0.03 micron^2^ (n = 323 lamellar bodies, 3 cats) versus 0.40±0.02 micron^2^ (n = 250 lamellar bodies, 3 cats) for wild type feline (*P*<0.05). Measurement of the lung lipid content (Fig. 10A) demonstrated a 60% increase in phospholipid and a striking 3.5-fold enrichment in cholesterol. Although the size of the lungs precluded an accurate assessment of the total surfactant lipid, the cholesterol to phospholipid ratio showed a 2.5-fold enrichment in cholesterol over normal feline surfactant (cholesterol/phospholipid ratio: wild type = 0.11±0.03, n = 4; NPC1 mut = 0.28±0.02, n = 3). The lamellar body phospholipid content did not change while the cholesterol content was elevated by 7-fold (Fig. 10B). Thus, the NPC1 mutant feline demonstrated lung disease with pathological manifestations comparable to those seen in mice.

## Discussion

Our study, using histological, immunological and biochemical procedures, demonstrates that the absence of NPC1 and NPC2 proteins in the lungs of mutant mice on a BALB/c background results in lung disease. Pathologic characteristics at the light microscopic level included thickened intra-alveolar septae, nests of foamy alveolar macrophages and vacuolar-filled circulating leukocytes. Electron microscopic analysis revealed excess surfactant in the alveolar space and capillary endothelial cells with enlarged vacuoles. The type II cells were shown to contain autophagosomes and lamellar bodies of abnormal sizes. Many of the lamellar bodies in NPC1 type II cells were enlarged and the majority in NPC2 type II cells were small. Cholesterol accumulation, a characteristic of the disease in other organ systems, also was found in the various components of the lung surfactant system. In both the NPC1 and NPC2 mutant mice, the lung tissue, isolated lamellar bodies, surfactant, and alveolar macrophages showed greater that 2-fold enrichment in cholesterol. In conjunction with this change, the phospholipid content of the tissue and cells also increased with a rise in SP-A content in lung and surfactant consistent with alveolar lipidosis and lipoproteinosis.

Initial reports found NPC1 and NPC2 mutant mice to be essentially identical using criteria such as accumulation of lipids in brain and liver [26]. Likewise, similar pulmonary abnormalities were seen in the lungs of NPC1 and NPC2 mutant mice such as accumulation of foamy, cholesterol-filled macrophages in the alveolar space [2], [6]. However, the lung phenotype is thought to be more severe in the NPC2 mice compared to that reported for NPC1 due to the lack of proteinosis in NPC1 mice, based on histological analysis [37]. Our results, on the other hand, demonstrated that alveolar lipidosis and other pathological changes were at least as severe in the NPC1 mutant mouse compared to the NPC2 hypomorph mouse. The main difference between the two types of NPC disease was the changes in lamellar body size. The more rapid progression of the disease in the NPC1 mouse versus the NPC2 mutant mice has been well documented and serves as a model for the severe infantile onset form of the human disease [26], [38].

The feline NPC1 disorder, which progresses more slowly, is felt to be an excellent model for the human pathogenesis [9]. Niemann-Pick disease type C felines harbor a single base substitution (2864G-C) resulting in an amino acid change from cysteine to serine (C955S) [9]. The cats demonstrate lysosomal storage disease with hepatic and neurological manifestations including lipid-laden macrophages in the spleen and bone marrow and unesterified cholesterol accumulation in skin fibroblasts [10]. In addition, as shown here, feline NPC1 disease also demonstrated a similar lung phenotype as the Npc1^nih/nih^ mutant mice. There was thickening of the alveolar walls, foamy capillary leukocytes, and lipid-filled macrophages in the alveolar space. In addition, the feline NPC1 type II cells contained enlarged (occasionally giant) lamellar bodies and lipid-enriched lamellar bodies, surfactant and lung tissue. Thus, both mice and feline animal models are suitable for studies of disease progression and pulmonary complications related to loss of a functioning NPC pathway.

We describe previously uncharacterized dysfunctions in type II cells of the NPC1 and NPC2 mutant mice that may help to clarify their contribution to the elevated surfactant cholesterol phenotype associated with Niemann-Pick type C. First, the surfactant storage organelles, the lamellar bodies, have significantly elevated phospholipid and cholesterol content, which correlate to elevated phospholipid and cholesterol in the surfactant. In support of this data, immunocytochemistry directly demonstrated excess cholesterol accumulated within lamellar bodies of type II cells isolated from the NPC mutant mice. Second, there were anomalies in the lamellar body size in the type II cells from both mutant mice: enlarged lamellar bodies in NPC1 mutant mice, mirrored in the NPC1 mutant felines; and small lamellar bodies in NPC2 mutant mice. The relationship between lamellar body size an pulmonary abnormalities is not yet clear although giant lamellar body formation was associated with impaired surfactant secretion in a mouse model of Hermansky-Pudlak syndrome (HPS) [39]. Thirdly, surfactant phospholipid degradation was inhibited, presumably due to an effect on type II cells as they play a major role in the degradation of surfactant PC [40]. What other alterations in surfactant in surfactant metabolism occur remains to be determined. Our studies highlight the importance of addressing lung epithelial function as a potential underlying cause of lung involvement in NPC disease.

Lamellar body size is an important indication of type II cell health since lamellar body size is altered in a number of genetic diseases such as ABCA3 deficiency and HPS [39], [41], [42]. We have shown the presence of NPC1 and NPC2 in these lysosome-related organelles where they may play a role in the regulation of lamellar body surfactant cholesterol [19]. It is tempting to speculate that the Niemann-Pick C proteins also may play a role in lamellar-body genesis. Lamellar bodies originate as multivesicular bodies that rearrange their internal phospholipid-containing membranes to form larger lamellar bodies [43]. Although the cholesterol content of the lamellar body from NPC mutant mice is high, it is unlikely to be the reason for an enlarged organelle as cholesterol only represents a small fraction of the total lipid. Since two lamellar body populations (small and medium) exist in normal mice while only one population (small) is found in the NPC2 mutant mice, NPC2 may play a role in the formation or maintenance of the medium sized lamellar bodies through its fusinogenic properties [44]. NPC2, located within the lamellar body, may be required for membrane rearrangement of multivesicular bodies, a necessary precedent for formation of larger “mature” lamellar bodies while NPC1 could interact with NPC2 and limit fusion. Thus, the absence of NPC2 might result in a lack of fusion and smaller lamellar bodies while the absence of NPC1 would result in unchecked fusion and large lamellar bodies.

The absence of NPC1 protein in mice negatively affected pulmonary function, where inspiratory capacity, elastance and hysterisivity were increased in the diseased lungs [37]. The mechanisms whereby elevation in lung and surfactant cholesterol can result in disruption in the physiology and in the turnover of surfactant are unknown but might include: First, alterations in surfactant properties. Cholesterol is a critical yet understudied component of surfactant that contributes to surface viscosity, such that excess cholesterol interferes with surfactant function [2], [12], [45]. Second, membrane properties may be altered due to the inability of cholesterol to exit the lysosomes and, thus, be unable to reach the plasma membrane, detrimentally affecting plasma membrane lipid rafts, surfactant secretion and endocytic vesicular uptake of surfactant. Finally, free cholesterol accumulation has been implicated in the intensification of oxidative stress and apoptosis, both of which would lead to dysfunction of pneumocyte metabolism [46].

In summary, we have identified defects in surfactant homeostasis in both NPC1 and NPC2-mutant mice including elevated surfactant phospholipid and cholesterol, elevated lamellar body phospholipid and cholesterol, and abnormal lamellar body sizes. Many of these pathologic changes were noted in the NPC1 mutant feline pulmonary system as well. The work underscores the importance of understanding the regulation of cholesterol trafficking to maintain proper pulmonary function. These studies elucidate novel characteristics of lung disease progression in Niemann-Pick C1 and C2, and demonstrate that epithelial cell dysfunctions can be a contributing source for lung disease.

## References

[1] Rosenbaum AI, Maxfield FR (2011) Niemann-Pick type C disease: molecular mechanisms and potential therapeutic approaches. Journal of neurochemistry 116: 789–795. 20807315 10.1111/j.1471-4159.2010.06976.x PMC3008286

[2] Griese M, Brasch F, Aldana VR, Cabrera MM, Goelnitz U, et al (2010) Respiratory disease in Niemann-Pick type C2 is caused by pulmonary alveolar proteinosis. Clin Genet 77: 119–130. 20002450 10.1111/j.1399-0004.2009.01325.x

[3] Meiner V, Shpitzen S, Mandel H, Klar A, Ben-Neriah Z, et al (2001) Clinical-biochemical correlation in molecularly characterized patients with Niemann-Pick type C. Genetics in medicine : official journal of the American College of Medical Genetics. 3: 343–348. 10.1097/00125817-200109000-00003 11545687

[4] Millat G, Chikh K, Naureckiene S, Sleat DE, Fensom AH, et al (2001) Niemann-Pick disease type C: spectrum of HE1 mutations and genotype/phenotype correlations in the NPC2 group. Am J Hum Genet 69: 1013–1021. 11567215 10.1086/324068 PMC1274348

[5] Vanier MT (2010) Niemann-Pick disease type C. Orphanet journal of rare diseases. 5: 16. 10.1186/1750-1172-5-16 PMC2902432 20525256

[6] Manabe T, Yamane T, Higashi Y, Pentchev PG, Suzuki K (1995) Ultrastructural changes in the lung in Niemann-Pick type C mouse. Virchows Archiv : an international journal of pathology 427: 77–83. 7551349 10.1007/BF00203741

[7] Liu B, Ramirez CM, Miller AM, Repa JJ, Turley SD, et al (2010) Cyclodextrin overcomes the transport defect in nearly every organ of NPC1 mice leading to excretion of sequestered cholesterol as bile acid. J Lipid Res 51: 933–944. 19965601 10.1194/jlr.M000257 PMC2853461

[8] Morris MD, Bhuvaneswaran C, Shio H, Fowler S (1982) Lysosome lipid storage disorder in NCTR-BALB/c mice. I. Description of the disease and genetics. Am J Pathol 108: 140–149. 6765731 PMC1916074

[9] Somers KL, Royals MA, Carstea ED, Rafi MA, Wenger DA, et al (2003) Mutation analysis of feline Niemann-Pick C1 disease. Mol Genet Metab 79: 99–103. 12809639 10.1016/s1096-7192(03)00074-x

[10] Brown DE, Thrall MA, Walkley SU, Wenger DA, Mitchell TW, et al (1994) Feline Niemann-Pick disease type C. Am J Pathol. 144: 1412–1415. PMC1887453 8203477

[11] King RJ, Clements JA (1972) Surface active materials from dog lung. II. Composition and physiological correlations. American Journal of Physiology 223: 715–726. 5068620 10.1152/ajplegacy.1972.223.3.715

[12] Veldhuizen R, Nag K, Orgeig S, Possmayer F (1998) The role of lipids in pulmonary surfactant. Biochim Biophys Acta 1408: 90–108. 9813256 10.1016/s0925-4439(98)00061-1

[13] Gunasekara L, Schurch S, Schoel WM, Nag K, Leonenko Z, et al (2005) Pulmonary surfactant function is abolished by an elevated proportion of cholesterol. Biochimica et biophysica acta 1737: 27–35. 16216549 10.1016/j.bbalip.2005.09.002

[14] Vockeroth D, Gunasekara L, Amrein M, Possmayer F, Lewis JF, et al (2010) Role of cholesterol in the biophysical dysfunction of surfactant in ventilator-induced lung injury. American journal of physiology Lung cellular and molecular physiology 298: L117–125. 19897745 10.1152/ajplung.00218.2009

[15] Leonenko Z, Gill S, Baoukina S, Monticelli L, Doehner J, et al (2007) An elevated level of cholesterol impairs self-assembly of pulmonary surfactant into a functional film. Biophysical journal 93: 674–683. 17483162 10.1529/biophysj.107.106310 PMC1896251

[16] Hass MA, Longmore WJ (1979) Surfactant cholesterol metabolism of the isolated perfused rat lung. Biochim Biophys Acta 573: 166–174. 582287 10.1016/0005-2760(79)90183-8

[17] Carstea ED, Morris JA, Coleman KG, Loftus SK, Zhang D, et al (1997) Niemann-Pick C1 disease gene: homology to mediators of cholesterol homeostasis. Science 277: 228–231. 9211849 10.1126/science.277.5323.228

[18] Naureckiene S, Sleat DE, Lackland H, Fensom A, Vanier MT, et al (2000) Identification of HE1 as the second gene of Niemann-Pick C disease. Science 290: 2298–2301. 11125141 10.1126/science.290.5500.2298

[19] Roszell BR, Tao JQ, Yu KJ, Huang S, Bates SR (2012) Characterization of the Niemann-Pick C pathway in alveolar type II cells and lamellar bodies of the lung. Am J Physiol Lung Cell Mol Physiol 302: L919–932. 22367786 10.1152/ajplung.00383.2011 PMC3362154

[20] Infante RE, Wang ML, Radhakrishnan A, Kwon HJ, Brown MS, et al (2008) NPC2 facilitates bidirectional transfer of cholesterol between NPC1 and lipid bilayers, a step in cholesterol egress from lysosomes. Proc Natl Acad Sci U S A 105: 15287–15292. 18772377 10.1073/pnas.0807328105 PMC2563079

[21] Deffieu MS, Pfeffer SR (2011) Niemann-Pick type C 1 function requires lumenal domain residues that mediate cholesterol-dependent NPC2 binding. Proceedings of the National Academy of Sciences of the United States of America 108: 18932–18936. 22065762 10.1073/pnas.1110439108 PMC3223457

[22] Kwon HJ, Abi-Mosleh L, Wang ML, Deisenhofer J, Goldstein JL, et al (2009) Structure of N-terminal domain of NPC1 reveals distinct subdomains for binding and transfer of cholesterol. Cell 137: 1213–1224. 19563754 10.1016/j.cell.2009.03.049 PMC2739658

[23] Du X, Kumar J, Ferguson C, Schulz TA, Ong YS, et al (2011) A role for oxysterol-binding protein-related protein 5 in endosomal cholesterol trafficking. J Cell Biol 192: 121–135. 21220512 10.1083/jcb.201004142 PMC3019559

[24] Ramirez CM, Liu B, Taylor AM, Repa JJ, Burns DK, et al (2010) Weekly cyclodextrin administration normalizes cholesterol metabolism in nearly every organ of the Niemann-Pick type C1 mouse and markedly prolongs life. Pediatr Res 68: 309–315. 20581737 10.1203/PDR.0b013e3181ee4dd2 PMC3065173

[25] Loftus SK, Morris JA, Carstea ED, Gu JZ, Cummings C, et al (1997) Murine model of Niemann-Pick C disease: mutation in a cholesterol homeostasis gene. Science 277: 232–235. 9211850 10.1126/science.277.5323.232

[26] Sleat DE, Wiseman JA, El-Banna M, Price SM, Verot L, et al (2004) Genetic evidence for nonredundant functional cooperativity between NPC1 and NPC2 in lipid transport. Proc Natl Acad Sci U S A 101: 5886–5891. 15071184 10.1073/pnas.0308456101 PMC395893

[27] Ward S, O’Donnell P, Fernandez S, Vite CH (2010) 2-hydroxypropyl-beta-cyclodextrin raises hearing threshold in normal cats and in cats with Niemann-Pick type C disease. Pediatr Res 68: 52–56. 20357695 10.1203/PDR.0b013e3181df4623 PMC2913583

[28] Bortnick AE, Favari E, Tao JQ, Francone OL, Reilly M, et al (2003) Identification and characterization of rodent ABCA1 in isolated type II pneumocytes. Am J Physiol Lung Cell Mol Physiol 285: L869–878. 12909583 10.1152/ajplung.00077.2003

[29] Chander A, Johnson RG, Reicherter J, Fisher AB (1986) Lung lamellar bodies maintain an acidic internal pH. The Journal of biological chemistry 261: 6126–6131. 3700387

[30] Lowry OH, Rosebrough NJ, Farr AL, Randall RJ (1951) Protein measurement with the Folin phenol reagent. J Biol Chem 193: 265–275. 14907713

[31] Bartlett GR (1959) Phosphorus assay in column chromatography. J Biol Chem 234: 466–468. 13641241

[32] Bligh EG, Dyer WJ (1959) A rapid method of total lipid extraction and purification. Can J Med Sci 37: 911–917. 10.1139/o59-099 13671378

[33] Jain D, Dodia C, Bates SR, Hawgood S, Poulain FR, et al (2003) SP-A is necessary for increased clearance of alveolar DPPC with hyperventilation or secretagogues. American journal of physiology Lung cellular and molecular physiology 284: L759–765. 12676766 10.1152/ajplung.00200.2002

[34] Mason RJ, Nellenbogen J, Clements JA (1976) Isolation of disaturated phosphatidylcholine with osmium tetroxide. Journal of lipid research 17: 281–284. 932560

[35] Zhang M, Strnatka D, Donohue C, Hallows JL, Vincent I, et al (2008) Astrocyte-only Npc1 reduces neuronal cholesterol and triples life span of Npc1−/− mice. Journal of neuroscience research 86: 2848–2856. 18500759 10.1002/jnr.21730 PMC2634300

[36] Gurel O, Ikegami M, Chroneos ZC, Jobe AH (2001) Macrophage and type II cell catabolism of SP-A and saturated phosphatidylcholine in mouse lungs. American journal of physiology Lung cellular and molecular physiology 280: L1266–1272. 11350807 10.1152/ajplung.2001.280.6.L1266

[37] Muralidhar A, Borbon IA, Esharif DM, Ke W, Manacheril R, et al (2011) Pulmonary function and pathology in hydroxypropyl-beta-cyclodextin-treated and untreated Npc1/mice. Mol Genet Metab 103: 142–147. 21459030 10.1016/j.ymgme.2011.03.001 PMC3107736

[38] Maue RA, Burgess RW, Wang B, Wooley CM, Seburn KL, et al (2012) A novel mouse model of Niemann-Pick type C disease carrying a D1005G-Npc1 mutation comparable to commonly observed human mutations. Hum Mol Genet 21: 730–750. 22048958 10.1093/hmg/ddr505 PMC3263988

[39] Guttentag SH, Akhtar A, Tao JQ, Atochina E, Rusiniak ME, et al (2005) Defective surfactant secretion in a mouse model of Hermansky-Pudlak syndrome. Am J Respir Cell Mol Biol 33: 14–21. 15790974 10.1165/rcmb.2004-0293OC PMC2715302

[40] Fisher AB (1998) Lung Surfactant Clearance and Cellular Processing. In: Rooney SA, Landes RG, editors. Lung surfactant: cellular and molecular processing. Austin, TX: Landes Bioscience. 165–189.

[41] Besnard V, Matsuzaki Y, Clark J, Xu Y, Wert SE, et al (2010) Conditional deletion of Abca3 in alveolar type II cells alters surfactant homeostasis in newborn and adult mice. American journal of physiology Lung cellular and molecular physiology 298: L646–659. 20190032 10.1152/ajplung.00409.2009 PMC2867401

[42] Nakatani Y, Nakamura N, Sano J, Inayama Y, Kawano N, et al (2000) Interstitial pneumonia in Hermansky-Pudlak syndrome: significance of florid foamy swelling/degeneration (giant lamellar body degeneration) of type-2 pneumocytes. Virchows Archiv : an international journal of pathology 437: 304–313. 11037352 10.1007/s004280000241

[43] Weaver TE, Na CL, Stahlman M (2002) Biogenesis of lamellar bodies, lysosome-related organelles involved in storage and secretion of pulmonary surfactant. Semin Cell Dev Biol 13: 263–270. 12243725 10.1016/s1084952102000551

[44] Abdul-Hammed M, Breiden B, Adebayo MA, Babalola JO, Schwarzmann G, et al (2010) Role of endosomal membrane lipids and NPC2 in cholesterol transfer and membrane fusion. J Lipid Res 51: 1747–1760. 20179319 10.1194/jlr.M003822 PMC2882726

[45] Orgeig S, Daniels CB (2001) The roles of cholesterol in pulmonary surfactant: insights from comparative and evolutionary studies. Comp Biochem Physiol A Mol Integr Physiol 129: 75–89. 11369535 10.1016/s1095-6433(01)00307-5

[46] Lee W, Xu M, Li Y, Gu Y, Chen J, et al (2011) Free cholesterol accumulation impairs antioxidant activities and aggravates apoptotic cell death in menadione-induced oxidative injury. Archives of biochemistry and biophysics 514: 57–67. 21843500 10.1016/j.abb.2011.07.014

